# Raising the ‘environmental question’ in social work in Canada and Scotland

**DOI:** 10.1177/00208728221094415

**Published:** 2022-05-18

**Authors:** Tina E Wilson, Heather Lynch, Verena K Fisch

**Affiliations:** Glasgow Caledonian University, UK; Glasgow Caledonian University, UK; Glasgow Caledonian University, UK

**Keywords:** Comparative studies, environment, professional education, social change, social ecology, social work

## Abstract

This article contributes a comparative review of social work in Canada and Scotland to international conversations about social work and the environment. The ‘environmental question’ of the 21st century is a radical challenge to social work developed in relation to the ‘social question’ of the 19th century. Work to begin to include the natural environment within high-income state social work can expect to encounter established infrastructures of thinking and doing that will be difficult to shift. We, therefore, compare guiding social work policy documents and identify points of tension that are likely to be shared across wealthy national contexts.

## Introduction

The question of the environment, posed by environmentalists with increasing urgency since the 1950s, became unavoidable on 8 October 2018, when the UN Intergovernmental Panel on Climate Change warned that catastrophic climate change will occur by the end of the present century (IPCC, 2018, 2021). The IPCC (2021) report has only reinforced the imperative for global leaders to act to reduce the scale of the unfolding disasters. This major generational shift pressures social work to reimagine research and education, policy and practice, for a world with problems of a rather different scale and foci than what has typically been captured by the “social question” that founds our loosely shared project.

Social work is certainly not alone in this work, as evidenced by an environmental turn occurring across the university, with many disciplines now looking to the natural sciences for resources with which to understand, imagine, and engage rapidly changing environments and their proliferating effects. This turn toward the natural environment is similarly visible in the social work literature both as a growing ‘sub-field’ inclusive of explicit value-theoretical agendas and as a more general topic of research (Krings et al., 2020), and in broader calls to revisit how social work understands the environment (Boetto, 2019; Bozalek and Pease, 2021; Liu and Flynn, 2021). However, the social work response to the question of environment remains niche and in development. For example, a survey of literature shows that growth interest is ‘limited to specific geographical regions and topics’ (Krings et al., 2020: 275). Given the urgency of environmental changes, it is essential to understand the specific challenges for social work in different historical and regulatory contexts.

Our point of departure for this article is that state-sponsored professionalized social work in high-income nations faces challenges in reorienting to begin to explicitly include the natural environment within our remit because we are established assemblages of interests and institutions developed in conversation with the modern ‘social question’ (see, for example, Chambon, 2013; Lorenz, 2014, 2016; Philp, 1979). Of note, changes within social work are unlikely to be synchronous. For example, a review of social work codes of ethics in Australia, the United Kingdom, and the United States concluded environmental sustainability is not in fact a perceived professional concern in these national contexts (Bowles et al., 2018). At the same time, the non-human environment is included within the Grand Challenges initiative in the United States (Kemp et al., 2018), and the American Council on Social Work Education (CSWE) has published an extensive curricular guide cross-mapping an environmental justice framework onto the existing national competency framework (CSWE, 2020). In turn, an analysis of the limited available Anglo social work curriculum literature identifies the environment or ‘green’ social work as one of four department- or curriculum-level theoretical orientations (along with critical postmodernism, critical race-structural, and Indigenous-decolonial orientations) (Cox et al., 2021). This said, a review of courses taught in Australia found attention to the environment is uneven across universities, concluding that social work education lags behind the published scholarship (Harris and Boddy, 2017).

The present article contributes to this variegated, evolving picture a comparative review of core social work policy documents in Canada and Scotland for the extent to which the natural environment is acknowledged or foregrounded. We begin with an overview of our approach and the policies examined, briefly outline the historical and institutional infrastructures of each of the two state-based social works, and then focus on the natural environment as it is articulated within the reviewed guiding documents. For the discussion, we consider three interrelated challenges evident in different ways in the two state social works examined. Overall, the article contributes to the ongoing inter- and trans-generational work of shifting established configurations of the profession in light of the ways in which the emerging ‘environmental question’ radically destabilizes the existing configuration of social work built around the ‘social question’ of modern political states.

## Comparing social work in Canada and Scotland

Comparison puts situated views into conversation so that we can more readily perceive the regional and historical nature of the ideas and infrastructures that comprise particular ways of life (Haraway, 1988). Comparative *research* contrasts two logically relatable entities or units of analysis to theorize processes of social change (Lange, 2014). For this article, we compare two high-income state social works – Canada and Scotland – for the extent to which the environment is acknowledged and foregrounded as a means to consider the challenges and affordances of different but linked historical configurations. We focus on guiding policy because these ‘fundamental documents’ organize perceptions of what matters (Boetto, 2019: 146). Of note, the curriculum standards of both countries have been recently revised, providing an up-to-date overview of current locally perceived problematics. Canada and Scotland are also the locations in which the authors live and work, and our conversations have regularly and productively turned to comparisons of our respective contexts as a way into broader questions about social work as a loosely shared project in a rapidly changing world.

Although conceptually problematic (a point we will return to in the discussion), for the purpose of this article, we define environment as the ‘natural environment’, that is, animals (excluding human beings) and plants and fungi, land and water and air. We began our comparison by identifying key institutional bodies in each of the two contexts and their guiding policy documents. We then searched for ‘environment’ and ‘natur*’ in each of the documents, reviewed returned hits, and extracted those referring to the natural environment for closer consideration. Table 1 includes a list of the 10 policy documents reviewed, the number of returned keyword hits, and the number of those returned hits referencing the natural environment.

**Table 1.** Document review.

Search terms: ‘environment’ and ‘natur*’ Inclusion criteria: reference to the natural environment Data collection: publicly available documents retrieved online June 2021			
Document		# Hits	# Included
Canada	1. CASW-ACTS (2005a) Code of Ethics	6	1
	2. CASW-ACTS (2005b) Guidelines for Ethical Practice	18	3
	3. CASW-ACTS (2020) Scope of Practice Statement	2	0
	4. NCCSWR-CCORTS (2012) Entry-Level Competency Profile for the Social Work Profession in Canada	2	0
	5. CASWE-ACFTS (2021) Education Policies and Accreditation Standards for Canadian Social Work Education	16	10
Scotland	1. SSSC (2003) Rules for Social Work Training 2003	0	0
	2. SSSC (2016) Codes of Practice for Social Service Workers and Employers	0	0
	3. Scottish Government (2017) Health and Social Care Standards	16	2
	4. SSSC (2018) Handbook for Quality Assurance and Enhancement	0	0
	5. SSSC (2019) Standards for Social Work Education in Scotland	17	0

In the next sections, we provide an outline of professional social work in Canada and Scotland, and then turn to the included text extracts to discuss the extent to which the natural environment is acknowledged or foregrounded.

## Social work in Canada

In Canada, social work is reproduced through university-based education and regulated by provincial-level professional associations. There are 43 universities providing accredited social work education in Canada, and student enrollment continues to expand. For example, in the 2008–2009 school year, 10,666 students were enrolled in undergraduate and master’s level social work programmes, increasing to 15,484 students in 2019–2020 (CASWE-ACFTS, 2019). Some social workers work for government ministries or in government-run welfare services. Most, however, work for non-profit community organizations or in ministry-mandated organizations (arm’s-length but funded by and reporting to the government). There are also a range of private or for-profit organizations providing supported housing and long-term care. The helping profession and para-professional landscapes are broad, inclusive of a range of education programmes and job titles including youth worker, social service worker, and personal support worker. The title ‘social worker’ is restricted to those registered with their provincial association, and graduates of social work education may in some cases only register or maintain registration if their employer requires it (for historical overviews of social work in Canada, see Chapman and Withers, 2019; Jennissen and Lundy, 2011).

There are three major federal-level social work bodies in Canada – profession, regulation, and education. Founded in 1926, the Canadian Association of Social Workers–Association canadienne des travailleuses et travailleurs sociaux (CASW-ACTS) is the national professional association, publishing the Code of Ethics (CASW-ACTS, 2005a), Guidelines for Ethical Practice (CASW-ACTS, 2005b), and Scope of Practice Statement (CASW-ACTS, 2020). Founded in 2009, the National Canadian Council of Social Work Regulators–Conseil canadien des organismes de règlementation en travail social (NCCSWR-CCORTS) authors the *Entry-level Competency Profile for the Social Work Profession in Canada* (NCCSWR-CCORTS, 2012) (for background, see Campbell, 2015). The final national body, the Canadian Association for Social Work Education–Association canadienne pour la formation en travail social (CASWE-ACFTS), was established (under a different name) in 1954 and is the membership organization for social work educators, combining the priorities of both professional and academic education. CASWE-ACFTS has since 1973 accredited Canadian university-based social work education. Prior to this, social work education in Canada was accredited by the US education association.

Since 2012, CASWE-ACFTS (2021) has undertaken an involved process of reworking earlier policy and accreditation documents into the combined *Education Policies and Accreditation Standards for Canadian Social Work Education* (EPAS). This new guiding policy and standards document explicitly identifies the environment as 1 of 13 core learning outcomes to be included in social work education in Canada. Of note, this new consideration was preceded by 20 years of concerted effort to make the environment intelligible as a social work problematic (Coates and Gray, 2018).

## Social work in Scotland

Scotland is one of four countries that form the United Kingdom. Its parliament Holyrood, established in 1999, deals with a range of devolved matters including education, health and social care, and environment. While it has some powers to raise taxes, the main fiscal powers are reserved for the UK government along with foreign policy, trade, defence, and energy. The Kilbrandon Report (Scottish Office, 1964), the policy that supported the development of social work in Scotland, is considered more progressive, communitarian, and aware of social justice than English social policy (Brodie et al., 2008). However, a more recent review of social work in Scotland (Scottish Executive, 2006) has been critiqued for following a neoliberal agenda of increasing individualism (for a history of social work in Scotland, see Cree, 2018).

In contrast to Canada, Scottish social work, like that of the broader United Kingdom, developed as a state system rather than one built around the individual professional. Social work is as a consequence bound to local government interests rather than to a more autonomous profession project. In contrast to England, however, Scotland has not adopted an apprenticeship model of education (McCulloch, 2018), and all social workers must instead qualify through undergraduate or postgraduate university-based degree programmes. The university-based Scottish education system is thus comparable to that found in Canada. This said, it is a much smaller system with only nine higher education institutions providing social work education in contrast to Canada’s 43. Although post-Kilbrandon Scottish social work included community development work, the hollowing of the welfare state (Roberts and Devine, 2003) since has seen the loss of those roles in favour of so-called ‘high tariff cases’ which require statutory involvement.

There are three major social work bodies in Scotland. The first two are member-led organizations. Social Work Scotland (SSW) supports social work leadership, and the Scottish Association of Social Work (SASW) supports professional social workers. SASW is an arm of the UK-wide British Association of Social Work (BASW). They lobby government on emerging social work concerns and provide guidance for social work staff such as *The Anti-Poverty Practice Guide* (BASW, n.d.). Established in the early 2000s, the government’s Scottish Social Services Council (SSSC) has an extensive remit as it regulates the entire social work and social care workforce in Scotland. In contrast to the division found in Canada, the SSSC is the regulatory authority for *both* professional social work and social work education, publishing the *Rules for Social Work Training* (SSSC, 2003), *Codes of Practice for Social Service Workers and Employers* (SSSC, 2016), the *Handbook of Quality Assurance and Enhancement* (SSSC, 2018), and the *Standards in Social Work Education in Scotland* (SSSC, 2019). The central regulatory role of the SSSC has been identified as an impediment to the development of a social work professional identity in Scotland, as workers are subordinate to this governmental agenda (Simpson et al., 2020).

The *Standards in Social Work Education* (SISWE) is the central curricular lever. First introduced in 2003, SISWE was revised in 2019, but not significantly. These revisions were informed by a series of small studies carried out with students, new graduates (McCusker and Jackson, 2016), managers (Welch, 2015), and social work stakeholders (McCusker and McCulloch, 2016), which has arguably created something of a closed loop tuned to the perceived urgencies of current employment contexts. Overall, SISWE emphasizes skills development in assessing risk, managing intervention, and advocating for those in need. At the same time, university-based social work programmes must also meet the core mission and values of the larger university, and all Scottish universities make claims to a green agenda in their strategies (though this is highly variable in practice, see People and Planet University League, 2019). In this context, the University of Stirling has developed a short Continuing Professional Development programme in Disaster Management, which demonstrates environmental interest, albeit through a human-centred crisis response framework.

## Foregrounding the ‘natural environment’

While there are noteworthy differences in attention to the ‘natural environment’ in the two state contexts, both iterations of social work conceptualize nature and people as somehow distinct and, for the most part, treat the environment as the container for human action. The natural environment is acknowledged in two of the five reviewed Canadian policy documents and foregrounded as a core learning outcome in EPAS, the just-released revised curriculum standards (CASWE-ACFTS, 2021). This is in contrast to two minor references to the natural environment found in one of the five reviewed Scottish policy documents. See Table 2 for the text extracts and their source documents.

**Table 2.** Included text extracts.

Search terms: ‘environment’ and ‘natur*’ Inclusion criteria: reference to the natural environment Data collection: publicly available documents retrieved online June 2021	
**Scotland**	**Scottish Government Health and Social Care Standards (2)** ● Included under headline standard and outcome ‘1: I experience high quality of care and support that is right for me’, under the principle of wellbeing: ‘1.32. As a child, I play outdoors every day and regularly explore the natural environment’. (p. 7) ● Included under headline standard and outcome ‘5: I experience a high-quality environment if the organization provides the premises’, under the principle of wellbeing: ‘5.19 My environment has plenty of natural light and fresh air, and the lighting, ventilation and heating can be adjusted to meet my needs and wishes’. (p. 15)
**Canada**	**CASW-ACTS Code of Ethics (1)** Included as a principle under Value 2: Pursuit of Social Justice: ‘Social workers promote social development and environmental management in the interests of all people’. (p. 5) **CASW-ACTS Guidelines for Ethical Practice (3)** Included under Value 8: Ethical Responsibilities to Society: ● ‘Social workers advocate for change in the best interests of clients and for the overall benefit of society, the environment, and the global community’. (p. 24) ● ‘8.5 Advocate for the Environment 8.5.1 Social workers endeavour to advocate for a clean and healthy environment and advocate for the development of environmental strategies consistent with social work principles and practices’. (p. 25) **CASWE-ACFTS (2021) Education Policies and Accreditation Standards for Canadian Social Work Education (10)** ● Included in the organization’s vision statement: ‘Vision: CASWE-ACFTS envisions an economically, socially, and environmentally just world based on humanitarian and democratic ideals that demonstrate respect for the worth, agency, and dignity of all beings’. (p. 3) ● Included as one of 13 core learning outcomes: ‘9. Environmental Sustainability and Ecological Practice Social work students shall have opportunities to . . . a) understand the need to create ecologically sustainable communities, economies and natural and built environments, in which all life forms and eco-systems can survive and thrive; b) identify and challenge environmental injustice and racism, i.e. the inequitable burdens borne by those who are socially and economically marginalised in relation to environmental degradation and hazards; c) advance environmental sustainability across individual, organizational and professional contexts; and d) embrace the role of social workers in advocacy for public policies and social practices that will ensure ecological health and environmental sustainability at local, regional, national, and global levels’. (p. 16)

These minor references are found in the Scottish *Health and Social Care Standards*, which identify the importance to children of regular access to the ‘outdoors’ and to the ‘natural environment’ as spaces in which to ‘play’ and ‘explore’ (Scottish Government, 2017: 7). Those accessing health and social care can in turn expect the ‘environment’ of service will have ‘plenty of natural light and fresh air’ (Scottish Government, 2017: 15). In both of these instances, the natural environment is contextual background to the action of human wellbeing. In the Canadian case, the professional association’s *Code of Ethics* is organized into six values, with reference to ‘environmental management’ included within value 2: ‘Pursuit of Social Justice’ (CASW-ACTS, 2005a: 5). The professional association’s *Guidelines for Ethical Practice* are organized into eight areas of ethical responsibility, with reference to the natural environment found under area 8: ‘Ethical Responsibilities to Society’ (CASW-ACTS, 2005b: 24−25). Here, ‘[s]ocial workers endeavour to advocate for a clean and healthy environment and advocate for the development of environmental strategies consistent with social work principles and practice’ (CASW-ACTS, 2005b: 24−25). These two general affirmations of the environment as the backdrop to human life by the national professional association are newly amplified in EPAS, the just-released national education and accreditation policy.

EPAS 2021 foregrounds the natural environment as the ninth of 13 core learning outcomes – ‘Environmental Sustainability and Ecological Practice’. This learning outcome is articulated through four sub-clauses emphasizing ‘the need to create ecologically sustainable communities, economies and natural and built environments’, as well as the role of social workers in ‘challeng[ing] environmental injustice and racism’, ‘advance[ing] environmental sustainability’, and advocating for ‘ecological health and environmental sustainability at local, regional, national and global levels’ (CASWE-ACFTS, 2021: 16).

There are at least four recognizable frameworks suggested by the new Canadian accreditation standards: environmental-ecological *sustainability*, environmental *justice*, and ecological *health. Ecology*, not just *environment*, is also referenced. With this new learning objective, moreover, the remit of social work is expanded to include consideration for non-human life, that is, for ‘all life forms and eco-systems’ (CASWE-ACFTS, 2021: 16).

Of note, inclusion of the natural environment within the scope of professional social work education in Canada is something that will unfold over the next decade and more. Social work programmes in Canada are accredited for 8 years at a time (or for a shorter period if re-accredited with conditions) (CASWE-ACFTS, 2016). The new EPAS assessment framework includes a 2-year implementation period – until July 1, 2023 − during which programmes up for re-accreditation can choose if they use the old or the new education standards. As such, it will be roughly a decade before all university social work programmes are reviewed against the new policy. In contrast to Scotland, then, professional social work in Canada is at the beginning of a recognizable environmental turn.

## Social work and the ‘environmental question’

Our point of departure for this article is that the ‘environmental question’ of the 21st century radically challenges professional social work developed in response to the ‘social question’ of 19th century political states. We, therefore, conceptualize the environmental question as an uneven and in-process generational shift in perceptions of the possible and the desirable (Wilson, 2020). This shift requires those of us in human-centred and professionalized iterations of social work to revisit the scope and possibility of our established project, and to consider what might be involved in intentionally shifting the entangled interests and institutions that stabilize and reproduce what counts as intelligible and relevant. That is, what counts as ‘social work’.

The two high-income state social works considered here, Scotland and Canada, take different approaches to the natural environment. State-accredited and regulated social work in Scotland does not recognize the environment as a social work problematic, but rather treats the environment as one of many contextual factors for assessing risk factors related to human development and the adequacy of spaces of service provision. In Canada, the environment is given an affirmative nod by the professional association and is newly foregrounded as a core learning objective by the education association. In the Canadian case, the distribution of authority across profession, regulation, and education associations may permit a wider range of perspectives to emerge and remain distinct. For example, the Canadian regulator’s measurable competencies (NCCSWR-CCORTS, 2012) are similar to those found in Scotland (see SSSC, 2019) but encounter an alternative set of priorities in the national education association’s broader communitarian, social justice orientation. It may also be that the somewhat greater attention to the natural environment in settler state social work (i.e., Australia, Aotearoa New Zealand, Canada, and the United States) is due to the long-standing activism and scholarship of Indigenous peoples strategically negotiating liberal legal systems and settler nationalisms.

Along with their different infrastructures and foci, these two state social works can help us think about what are likely to be common challenges to incorporating greater consideration for the natural environment within established forms of professional social work organized around liberal democratic understandings of human welfare and around a largely urban and state-based or regional social collectivity. In this final section we therefore, consider three interrelated challenges evident in both reviewed cases as a contribution toward the collective work of shifting established configurations of professional social work in wealthy states.

The first challenge is the slippage that occurs when new ideas are introduced into an established field, and the consequent need to preserve the distinctiveness of the still-emerging environmental question so that it is not simply overwritten by the more established social question (Coates and Gray, 2018). We need to keep an eye on the networks of imaginaries we amplify through work centring the natural environment because infrastructures of thinking and doing, once established, become more difficult to shift. For example, in the Canadian policy reviewed here, human-centric sustainability, justice, and health frameworks, and a more metaphoric use of environment as location, anchor the new introduction of ecological complexity into social work education. The published literature shows similar slippage, with many authors writing about the environment in English language social work journals focused on natural disasters or crises, natural resources, and sustainable development (Krings et al., 2020). Environmental justice is often approached as similar to, or as an aspect of, social justice (Beltrán et al., 2016; Shajahan and Sharma, 2018), and environmental rights is treated as part of community development and political action (Androff et al., 2017). This translation of the less intelligible through the more intelligible, the increasingly perceived through existing investments, is a main way in which socio-political imaginaries shift over time and the importance of this work is not to be dismissed. It does, however, create a kind of noise that then becomes something to be navigated and refocused so that we also engage with more thoroughgoing challenges to established ways of life and to the perceived scope of social work.

This brings us to our second challenge, the ubiquitous treatment of the environment as metaphoric stage, context, or container for the action of human life, that is, the ‘human exceptionalism’ that centres human life as the focal figure against a less relevant and somewhat blurry or indistinct background (see discussions in Bell, 2012; Boetto, 2019; Bozalek and Pease, 2021; Coates and Gray, 2018; Jeffery, 2014). Here, social work can learn from migration and globalization scholars who problematize the long-standing ‘methodological nationalism’ of the social sciences (Wimmer and Glick Schiller, 2002). That is, the tendency to naturalize the political state, treating it as the ‘container’ within which human life occurs, and eliding synchronous happenings outside of, but also in entangled relation with, this unit of analysis and its perceived contents. Human exceptionalism operates similarly as a kind of ‘methodological anthropocentrism’, with consequences for how those of us in the applied social sciences perceive, imagine and practise. One major effect of this emphasis on the human in their ‘localized social’ (O’Brien, 2004) – on people in specific regional and state contexts – is that we are surprised when the ‘out of scope’ suddenly intrudes upon us. Case in point, the global COVID-19 pandemic is presented as a human war against the invader virus.

The methodological anthropocentrism of professional social work is highlighted in a recent review of 64 country-level codes of ethics that found ‘environment’ most often referred to social rather than the physical world (Liu and Flynn, 2021). In our work, this human focus is most overt in the Scottish case, where the natural environment is not in fact recognized as a social work problematic. The one noteworthy curricular innovation out of Stirling University is tuned to helping humans respond to crisis resulting from human-induced environmental harms. This focus on disaster is increasingly also found in the literate (see, for example, Alston and Chow, 2021), and takes an unabashed human perspective, as the disaster is experienced by human life. In contrast, a more thoroughgoing environmental turn is now in evidence in Canadian social work education – including acknowledgment of ‘all life forms and eco-systems’ (CASWE-ACFTS, 2021:16) – albeit nested within an established communitarian iteration of the human-prioritizing social question.

Environmental thought that rejects the Cartesian separation of mind and body provides a way into understanding some of the consequences of this habitual methodological anthropocentrism, tracing the ways in which human life cannot be separated from all life – not biologically (Crutzen and Stoermer, 2000), not socially (Chakrabarty, 2019; Ingold, 2013; Povinelli, 2016; Wolfe, 2013), and not materially (Bennett, 2010). Humans are microbial assemblages, dynamic swarms of microbiota within which human form might be recognized, but not absolutely defined. For example, debates around the Anthropocene (Crutzen and Stoermer, 2000) offer a view of human life from the vantage point of deep time. They trace ways in which human life is indelibly entangled with earth matter through its biological development and more recently as human activities and technologies leave their marks on the planet. Although there is a debate on the point when this started, there is no doubt that the carbon-fuelled industrial revolution of the 1800s is the main culprit. Technology that ushered in a wave of human progress also brought a raft of harms. This leads to the inescapable realization that human life cannot be separated from all life and cannot be a dominion within a dominion.

This brings us to our final interrelated challenge: the massive ethical implications of displacing the human as the central or primary concern in professional social work. In a broad sense, the social question that founded professional social work asks us to consider ‘what constitutes social bonds’ (Lorenz, 2016: 4), that is, how might humans live together relatively well. The answer given, however, changes over time and place (see Harrikari et al., 2014). Histories of the social question largely echo Enlightenment histories; the social question as a question of human nature and how societies might, therefore, go about advancing social progress (Lorenz, 2016). It evolved over time from concern for human freedom benchmarked against the foil of corrupt religious elites and then against corrupt monarchies, and by the end of the 19th century, the social question became a question of democracy benchmarked against the overt harms of industrial capitalism (Howerth, 1906). Here, inequality and poverty were problematized as a main impediment to social progress and stability, and the welfare state was developed as a pacifying solution (Kivisto, 2021). In the post-war welfare state, the social question shifted again, this time toward the problem of forms of human heterogeneity not addressed by the redistribution policies of the welfare state (Faist, 2021; Fraser and Honneth, 2003).

In the Canadian case, the social question has continued to shift through a range of corrective policies addressing various forms of group-stratified violence that is no longer perceived to be justified. This type of state-based corrective work is clearly visible in the marked groups identified in distinct learning outcomes in the revised Canadian EPAS – anti-Indigenous, anti-Black, and anti-Asian racisms; Francophone minorities; equity-seeking groups – alongside newly foregrounded concern for the natural environment. In the Scottish case, in contrast, rather than the corrective-inclusion work of Canadian liberal multiculturalism, the ‘social question’ more often revolves around a general loss of attention to community under neoliberal forms of government-run welfare provision (Butler-Warke et al., 2020; Elder-Woodward et al., 2015). That is, how neoliberal imaginaries disaggregate human subjects from the conditions in which they are formed and the consequent need to return to a more interconnected understanding of people and their circumstances.

Despite their different approaches to the natural environment, then, in both states, social work is founded in engagement with group-based stratification *within* the exceptional category of the ostensibly universal human subject (see Philp, 1979), that is itself nested *within* the exceptional category of the modern nation state. From an environmental viewpoint, however, the problem with the state-bounded liberal/communitarian, individual/community faultline that preoccupies so many of us is that it ignores the non-human world (see also Bozalek and Pease, 2021; Lynch, 2021). Moreover, social work’s guiding political coordinates of Left and Right, liberal individualism versus socialist communitarianism, share a devotion to industrialism – a philosophy of perpetual growth in production and human comfort – that largely ignores the environmental harms that follow from this way of life (Daggett, 2019; Dryzek, 2005; in social work see Bell, 2012).

In these ways, the environmental question of the 21st century is a radical challenge to social work developed in relation to the social question of 19th century nation states. As we have explored here in relation to Canada and Scotland, work to begin to include nature within the remit of a given iteration of state-based social work can expect to encounter historical infrastructures of thinking and doing that will be difficult to shift. In the Canadian case, the range of social work institutions and interests, along with Indigenous sovereignty movements, appears to have supported the emergence of greater concern for the natural environment, in contrast to the more centralized, government-regulated social work in Scotland. In the Scottish case, regaining community work as an intelligible form of social work may be required.

Based on this analysis, social work in high-income states will need to keep an eye on the ways in which the familiar – justice, sustainability – can assimilate the radically new and disruptive; on the ways in which habitual methodological nationalism and methodological anthropocentrism undermine shared dialogue toward the creation of larger alternative views; and on the cascading ethical implications of intentionally displacing modern conceptions of the human and the social from their established place as social work’s privileged objects. Pragmatically, social workers in direct practice can begin to incorporate the more-than-human world (Abram, 1996) into existing assessments and interventions (e.g., including attention to air quality). Classrooms remain important sites of collective wisdom, imagining, and inter-generational change (e.g., exploring how nature and the more-than-human are understood in the places people grew up, and considering how the climate crisis complicates inherited units of micro, meso, and macro practice). At the same time, there is a pressing need for programme-wide curricular revisions that engage the environmental question together with calls to decolonize the university and disrupt monoculturalism in our education and profession (Battiste, 2013; Bruyere et al., 2020 Shahjahan et al., 2022; TRCC, 2015). Western science is one of many, and the division between social and natural sciences is an historical and cultural convention. These programme-level changes will require significant resourcing and support from those in leadership roles. One strategy is to sponsor PhD and postdoctoral work in these areas. In turn, place-based research exploring more-than-human relations and climate adaptation, and historiographic research into how the social and the natural have been understood, combined, and divided in the historical development of modern social work are all important future directions for research.

## Conclusion

Broadly perceived questions-problems put divergent interests into relation, with unexpected effects. In professionalized iterations of social work, this has mainly revolved around the social question – that is, how to evoke some sort of normative stabilizing ‘us’ among mobile populations negotiating stratified challenges and affordances in particular times and places (see Chambon et al., 2015; Chapman and Withers, 2019; Köngeter, 2017). Evolving answers to the social question have included everything from colonial camps to urban workhouses, community playgrounds to nation states. These responses have over time led to massive, networked imaginaries crisscrossing political states, social institutions, and the division of knowledge labour within the university. Once built, these ways of life are extremely challenging to shift. Case in point, the two state-based social works considered here, along with the range of historical, regional, and international imaginaries, policies, and laws they each network with differently.

Raising the environmental question in social work is thus a question of how, from in the middle of group-stratified ways of life, we might learn to live in ways that create and displace less risk and harm onto other people, entities, and regions; how we might attempt to repair currently perceived harms; and also, how we might expand the scope of modern social work to include the more-than-human within understandings of the collective (Lynch, 2019).
